# Nonstrict and individual enhanced recovery after surgery (ERAS) in partial hepatectomy

**DOI:** 10.1186/s40064-016-3688-x

**Published:** 2016-11-25

**Authors:** Xingwei Xu, Yingbin Wang, Tao Feng, Xin Zhao, Yannian Liao, Wu Ji, Jieshou Li

**Affiliations:** 1 Jinling Hospital, Research Institute of General Surgery, Nanjing University, School of Medicine, Nanjing, 210002 Jiangsu Province People’s Republic of China; 2 General Surgery, General Hospital of Tisco Affiliated to Shanxi Medical University, Taiyuan, 030008 Shanxi Province People’s Republic of China

**Keywords:** Nonstrict, Individual, ERAS, Discharge

## Abstract

**Background:**

We aimed to evaluate postoperative recovery and short-term outcomes of patients undergoing partial hepatectomy managed with a nonstrict and individual enhanced recovery after surgery (ERAS) program.

**Methods:**

A retrospective analysis of 168 partial hepatectomy patients in our institution was included. The discharged day and the respective impact of element application throughout the duration were analyzed.

**Results:**

When all the required elements of ERAS were fully implemented, the median discharge day was 6. The more deviation occurred, the more delayed the patient discharged (*P* < 0.01). Preoperative ASA score, basic conditions of patients and ages were revealed closely associated with discharge day (*P* < 0.001). Without or an early removal of tubes and early oral feeding reduced hospital stay statistically (*P* < 0.01). Early discharge of patients (<3 days) did not show an increased complication incidence or readmission (*P* > 0.05).

**Conclusion:**

Nonstrict and individual use of ERAS in partial hepatectomy reduced postoperative length of stay without increasing complication rate. Our study proposes a modulation of ERAS according to the needs and acceptance of patients. In a word, better optionally required rather than mandatorily meet.

## Background

The concept of enhanced recovery after surgery (ERAS) was first introduced in colorectal surgery 15 years ago. Since then, ERAS strategy has been applied and adopted successful in other specialties areas, including urology, vascular and orthopedic surgery. ERAS refers to combining multimodal pathway including anesthesia, surgical, nursing and perioperative management to accelerate recovery, preserve body composition, and shorten discharge time without affecting morbidity. It also improves efficiency of hospital beds use and a decrease of hospital cost (Kim et al. 2012; Gouvas et al. 2009; French et al. 2009; Bosio et al. 2007).

Partial hepatectomy is still the most common treatment for liver tumor, and there are some non-randomized studies showing that ERAS significantly reduces length of hospital stay, lowers complication rates, and cuts total costs without any increase in mortality or readmission (Schultz et al. 2013; Hughes and McNally 2014). Neverless, major morbidity ranges from 17% in benign to 27% in malignant disease, with a mortality risk of up to 5%. The reality we found in clinical is that most patients could rarely often strictly meet all the elements of ERAS, while a strict ERAS requires high standards for clinical team and stringent inclusion criteria of the patients. Therefore, not attainable in every institution (Connor et al. 2013). Therefore, to verify whether a nonstrict and individual ERAS is feasible in patients of partial hepatectomy, we have decided to take this retrospective study to compare the short-term outcomes.

## Patients and methods

### Trial design

From January 2014 to July 2015, all patients between the ages of 16 and 75 years who underwent partial hepatectomy by laparoscopic procedures for liver cancer at the Department of Surgery, Jinling Hospital were considered to be included into the study. The inclusion criteria were: (1) elective partial hepatectomy for liver cancer or tumor; (2) no major concomitant surgical procedures, such as bowl, gastro or bile duct resection; (3) tumors either in the right or left hemiliver with the extent of partial hepatectomy being a hemihepatectomy or less; (4) Child–Pugh A/B liver function status; (5) without severe contraindications that not suited for ERAS (such as anticoagulant therapy).

Hepatectomy were all carried out by the same team of surgeons who had an experience of over 2000 hepatic resections. The study protocol was approved by the Ethics Committee of Jinling Hospital and Nanjing University. All clinical investigation has been conducted according to the principles expressed in the Declaration of Helsinki.

According to the literature and practical experience in our institution, giving up all traditional, not evidence-based measures, a brief total of 14 ERAS elements were concluded and listed as follow (Table 1). We avoided forcing each patient meet all elements. Instead, simply to calculate how many numbers of points each patient complied with, they were divided into four groups: (1) all-respected; (2) one point not respected, (3) 2–3 points not respected, (4) >3 points not respected (anyone who needed a re-insertion of tubes or fast was considered failed to respect element). The primary outcome measure was the discharge day after operation. Criteria for discharge were: pain sufficiently controlled by oral analgesics, a good recovery of liver function, an acceptable level of mobilization, tolerated solid food, no intravenous fluids, and no untreated surgical complications (Schultz et al. 2013).

**Table 1:** Nonstrict and individual ERAS program in our center

	ERAS element in our center
Day before surgery	
Preoperative information about ERAS	An inform of ERAS with good mood
Preoperative bowl preparation	No enema
Preoperative fasting	Normal oral nutrition until midnight; carbohydrate drink up to 2 h before surgery
Premedication	Omission as possible
Day of surgery	
Nasogastric tubes	Removed at the end of surgery
Anesthesia	General anesthesia combined with epidural anesthesia, with low dose of epidural analgesia in 2 days
Hypothermia	Prevention hypothermia
Fluid therapy	“Restrict fluid regimen”, avoid excessive i.v. fluids (CVP <5 mmHg), appropriate vasoconstrictor instead
Day after surgery	
Drainage of peritoneal cavity	No, or remove on day 1
Laxative	Start laxative (lactulose oral solution 30 mg) from day 1
Urinary catheter	No, or removed <24 h
Oral liquid take	Drink about 0.5 L liquid on day 1; at least 1 L liquid on day 2; normal diet from day 3
Postoperative analgesia	Flurbiprofen, tramadol in 2 days, followed with NSAID’s
Mobilization	Promote in bed on day 1, a minimum of four times per day; enforced mobilization from day 2

*NSAIDs* non-steroidal anti-inflammatory drugs

The factors which could influence the discharge day were identified and classified as following: (1) patient-related factors, (2) medical-related factors. The relevance between discharge day and each factor was generally measured. Outpatient appointment and telephone calls were made to see whether there were any complications or discomfort 30 days after operation, including the occurrence of readmission.

### Statistical analysis

The data on patients’ details, postoperative courses were respectively collected. Since the distribution of the dependent variable, hospital discharge, is abnormal and asymmetric, the presentation of the results is mainly based on the analysis of the median accompanied by quartile (Anastasiadis et al. 2013). Univariate analysis was performed using the Kruskal–Wallis test first and if there is a difference, then the Mann–Whitney *U* test is applied to identify between which of them we have difference. Comparison between discrete variables used the Chi square test and Fisher’s exact test. All analyses were performed using SPSS 11.0 software, *P* < 0.05 was considered statistically significant.

## Results

**Fig. 1:**
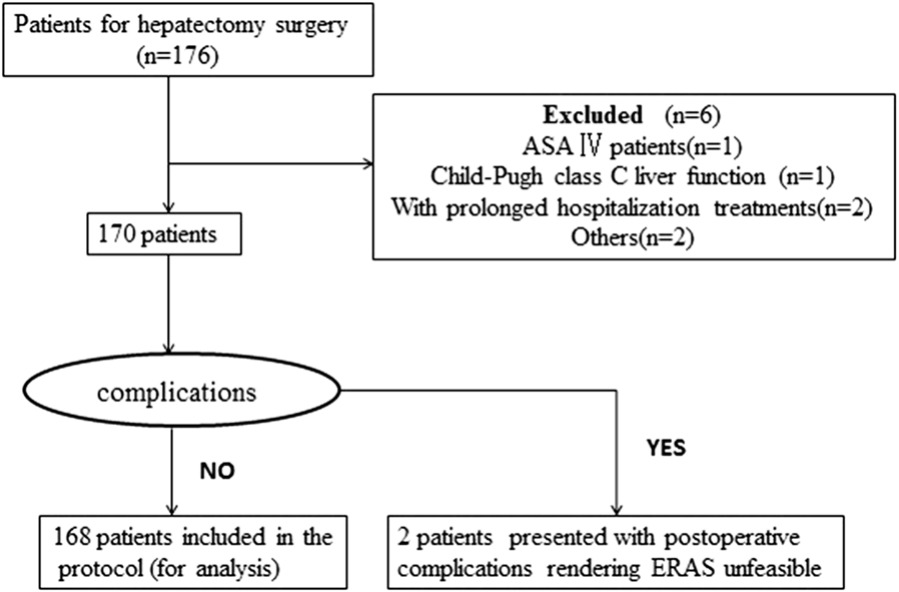
Flow chart

During this time period, there were 176 patients performed partial hepatectomy in our center. Finally, only 168 patients met the inclusion criteria and participated in this study (Fig. 1).

All patients had surgery in a high-volume hepatobiliary unit, Jinling Hospital. When all the required ten measures of ERAS fully implemented, the median discharge day was 3, compared with a median of 5 days for those >3 points not respected (*P* < 0.001). The more deviations occurred, the more delayed the patient discharged (Table 2).

**Table 2:** Nonstrict and individual ERAS of patients undergoing hepatectomy

	N	Discharge day med (P25–P75)	*P* value
All elements respected	64 (38.10)	3 (3–4)^a^	<0.001
1 point not respected	49 (29.17)	3 (3–4)	
2–3 points not respected	36 (21.43)	4 (4–6)	
>3 points not respected	19 (11.31)	5 (4–8)	

^a^ compared with those >3 points not respected

The basic patient-related factors, such as gender, age, ASA grade, BMI and preoperative complications, were grouped and assessed about their impact on discharge day as shown in Table 3. Patients who have vary in ASA, initial complication or age, had a significant statistical difference in median discharge day (*P* < 0.01). There was no significant difference in discharge between patients having a different preoperative diagnosis. Although without reaching statistic significance, patients having body mass index (BMI) inferior to 20 kg/m^2^ had a growing tendency towards prolonged hospitalization.

**Table 3:** Patient-related factors and their impact on discharge day

	N (%)	Discharge day med (P25–P75)	*P* value
Gender			
Male	83 (49.40)	4 (3–7)	0.592
Female	85 (50.60)	4 (3–6)	
Age			
16–35	15 (8.93)	4 (4–5)	<0.01
36–55	56 (33.33)	4 (3–6)	
56–75	81 (48.21)	6 (4–7)	
ASA			
1	65 (38.69)	4 (3–6)	<0.001
2	90 (53.57)	5 (3–7)	
3	13 (7.74)	7 (5–9)	
BMI			
<20	17 (10.12)	5 (5–7)	0.373
20–25	56 (33.33)	4 (4–5)	
25–30	84 (50.00)	5 (3–7)	
>30	11 (6.55)	5 (3–7)	
Pathology			
Colorectal metastases	23 (16.07)	4 (3–6)	0.503
Cholangiocarcinoma	27 (10.12)	5 (3–5)	
Gastrointestinal metastases	17 (13.10)	6 (3–7)	
Hepatocellular carcinoma	86 (27.38)	5 (4–7)	
Others	15 (8.93)	5 (3–7)	
Type			
Right hepatectomy	87 (51.79)	4 (3–5)	0.704
Left hepatectomy	66 (39.29)	4 (3–5)	
Segmentectomy	15 (8.93)	4 (3–6)	
Co-morbidities			
Cirrhosis	12 (7.14)	6 (5–9)	<0.001
Other	57 (33.93)	4 (3–6)	
No	99 (58.93)	4 (3–5)	

Discharge day based on medical-related factors (surgery, nasogastric, urine tube, drainage, and oral feeding) are shown in Table 4. When patients with temporary use or early removal of nasogastric tube, drainage, and urine catheter received, median discharge day all shortened significantly (*P* < 0.01, *P* < 0.001, *P* < 0.001, respectively), respectively. Early oral feeding and mobilization, usually aiming for recovery of bowel function, were encouraged with a growing tendency towards discharge day and reaching statistic significance.

**Table 4:** Medical related factors and their impact on discharge day

	N (%)	Discharge day med (P25–P75)	*P* value
Enema			
No	147 (87.50)	4 (3–6)	<0.001
Yes	21 (12.50)	6 (5–7)	
Fasting			
As guide	155 (92.26)	4 (3–5)	<0.001
No	13 (7.74)	5 (5–6)	
Nasogastric tube			
No	129 (76.79)	4 (3–6)	<0.01
Yes	39 (23.21)	5 (4–7)	
Drainage			
No	114 (67.86)	3 (3–5)	<0.001
Yes	54 (32.14)	5 (4–7)	
Urine cath			
No or remove in 24 h	146 (86.90)	4 (3–5)	<0.001
More than 24 h	22 (13.10)	6 (5–6)	
Anesthesia			
Complied to ERAS	141 (83.92)	4 (4–5)	0.440
Traditional	27 (16.07)	5 (5–6)	
Fluid therapy			
Complied to ERAS	137 (81.55)	4 (3–6)	0.355
Traditional	31 (18.45)	5 (4–6)	
Analgesia			
Complied to ERAS	140 (83.33)	4 (3–5)	0.268
Traditional	28 (16.67)	5 (4–6)	
Mobilization			
D1, four times per day	135 (80.36)	3 (3–5)	<0.001
Postponed	33 (19.64)	5 (4–7)	
Feeding			
D1	128 (76.19)	3 (3–5)	<0.001
More than D1	40 (23.81)	5 (4–8)	

After analyzing all data, we found that there were three readmissions in <3 days (early discharge), one for abnormal liver function, and other two for local infections. Complication incidence and readmission within the first postoperative month did not show significant difference in hepatectomy patients Thirty-day mortality was zero. Early discharge of patients did not result in significantly severe problems, as illustrated in Table 5 (*P* < 0.05) (15 patients got a series of complications, such as infection, intra-abdominal hemorrhage, abnormal liver function etc., ten returned for secondary treatment).

**Table 5:** Is discharge in 3 days safe in nonstrict and individual ERAS hepatectomy?

	With complications after discharge	Without complication	With readmission	Without readmission
Discharge ≤D3	5	51	3	53
Discharge >D3	10	102	7	105
*P* value	>0.05		>0.05	

All data in postoperative 30 days

## Discussion

According to the guidelines of ERAS programmes, a series of effective items to reduce surgical stress and accelerate recovery are usually used during perioperative period (Cerantola et al. 2013; Varadhan et al. 2010; Ansari et al. 2013). It has been widely demonstrated feasible and superior to traditional methods in different surgical fields. However, many of the principles of the multimodal pathway are derived from the colorectal ERAS and distinct differences exist, which may impede its implementation in HPB surgery (Hall et al. 2012). Besides, different understanding of the concept, variability of each institution’s practice, surgeons’ personal habits often makes ERAS “a key criterion, but various protocols” (Jones et al. 2013).

Under these conditions, the idea of soft and individual optimized ERAS has been raised, first in gastrointestinal surgery. Recently, a systematic review found substantial possibilities and advantages of optimizing ERAS in a more friendly way after an elective colorectal resection (Agrafiotis et al. 2013). It required the doctors and nurses to expand the ERAS inclusion criteria, deal affairs in a patient-friendly way based on their actual condition, and discontinue non-standard habits or mandatory practice. The implementation was proved easy accepted and not at the expense of increased rates of readmission, morbidity or mortality. Thus we adopt this idea in hepatobiliary surgery. A series of flexible elements were conducted for avoidance of reluctance; different items were introduced to each patient for appropriate extent; a better outcome finally gained and the importance and priority of each ERAS item evaluated.

Not surprisingly, the present study implied a tendency towards a shorter time of discharge in patients who have more degrees of compliance with ERAS items. As nearly half of cases selected were subjective but unwilling to unconditionally accept ERAS items, it is clear that this phenomenon reflects the extent of ERAS match and compliance as a cause–effect relationship to fast recovery and discharge, not due to a combination of an association that the healthier the patient, the earlier will he eat and get mobilized, but results of a positive correlation.

Patients’ nervous, fear and other negative emotions often occurs and can not be ignored (Higginson and Booth 2011). To this end, conducting any effective ways to shift patients’ insecure concerns and reduce worries is necessary and important, aiming to mitigate unequal information exchange and violent tendency between patients and doctors in China. A multidisciplinary team consisting surgeons, anesthetists and nurses, who can commit all individuals to meet tasks for ERAS, were formed and introduced (Ni et al. 2013). Patient decision aids such as printed documents and online information sources increase the involvement of patients in decision-making process and also increase the value of informed consent.

With the continuous development of anaesthetic, it would be possible to establish a “catheter free” protocol, especially in hepatobiliary surgery. Although there is RCT shows using epodural analgesia means low complication rate after major liver resection, a concern is the possible prolongation of prothrombin time which may delay catheter removal and increase administration of corrective blood products, and a risk factor for kidney failure due to hypotension (Sakowska and Docherty 2009; Kambakamba et al. 2015). Recently, several studies have suggested that intrathecal opiates are a suitable alternative to epidural analgesia and traditional morphine PCA (Kasivisvanathan et al. 2014; Revie et al. 2012).

Our results proved that “no tube” is an important criterion to get shorter hospital stays. Nasogastric tube is advised temporary use only in the condition of stomach gas, and removal at the end of surgery. Increased pulmonary complications and longer time to return of bowel function were observed in patients with routine nasogastric tube (Sapkota 2013; Pessaux et al. 2007).

The strongest evidence to omit routine prophylactic drainage after major abdominal surgery arises from a meta-analysis published in 2004 (Petrowsky et al. 2004). From then, there is a debate about the value and risk of prophylactic drainage (Kyoden et al. 2010). According to our clinical experiences, all patients abandoned to use drainage tubes as possible, or take an early removal, which was proposed with aims of early mobilization. Patients showed willingness to have earlier mobile as they could suffer from less pain and fewer tubes. Only two cases of 168 patients had mild biliary fistula, one had ascites. The incident hadn’t increased significantly. We believe active postoperative monitoring and ultrasound can be good alternative (Vlug et al. 2011).

We also summarize that fluid therapy is critical point during postoperative recovery. In this study, according to individual differences among patients and target-oriented principle, limited fluid therapy and early oral drinking followed with semifluid and enteral nutrition, can accelerate the recovery of intestinal, and reduce hospitalization time. We conclude that goal-directed fluid therapy especially balanced crystalloid solution at the end of hepatic resection and during the first 6 h enabled a faster restoration of circulating volume with reduction in complications. The use of hetastarches and colloids is not advised to increases the risks of renal dysfunction. However, patients of ASA IV were advised not to take nonstrict and individual ERAS as they often required additional treatment after surgery.

Early postoperative oral nutrition has been reported to reduce catabolism, lessen stress reaction, and decrease resting energy expenditure and postoperative complications such as nausea, vomiting, bloating or enteroparalysis (Srinivasa et al. 2012; Nygren et al. 2005). Most patients can eat normal food at day one after liver surgery, and we recommend early oral intake to accelerate resumption of bowel function.

Our study show that abandoning any outdated and dogmatic habits, rational application of multi-mode treatment, focusing individually is a prerequisite to make ERAS an optimal result. Non-strict, soft and optimized ERAS in hepatoectomy surgery plays an equal, not decreased effect in postoperative recovery. Gaining experience from evidence-based medicine and flexible application of ERAS, will achieve better clinical results.
